# 536. Evaluation of the Clinical Utility of Universal Polymerase Chain Reaction Testing

**DOI:** 10.1093/ofid/ofac492.589

**Published:** 2022-12-15

**Authors:** Joshua D Donkin, Rebecca P Emery, James Polega, Curtis J Behenna, Adam J Caulfield, Gordana Simeunovic

**Affiliations:** Spectrum Health / MSU College of Human Medicine, Rockford, Michigan; Spectrum Health / MSU, GRAND RAPIDS, Michigan; Spectrum Health/Michigan State University, Grand Rapids, Michigan; Ascension Medical Group / University of Oaklahoma, Tulsa, Oklahoma; Spectrum Health, Grand Rapids, Michigan; Spectrum Health, Michigan State College of Human Medicine, Grand Rapids, Michigan

## Abstract

**Background:**

Despite advances in microbiologic techniques, for patients with complex infections it often remains a challenge to identify the causative infectious pathogen. Traditional cultures (Cx) may fail to grow microorganisms due to inadequate sampling, prior antibiotic use, or the inherent insensitivity of culture methods for fastidious pathogens. Molecular tests allow for the detection of microbial nucleic acids directly from clinical specimens and do not require the presence of viable organisms for identification. Universal polymerase chain reaction testing (UPCR) is offered though the University of Washington Department of Laboratory Medicine and Pathology as a metagenomic approach using broad-range PCR primers followed by sequencing to hypothetically identify any pathogen present. The testing is composed of 3 separate tests for bacterial (BUPCR), fungal (FUPCR), and acid-fast (AFUPCR) organisms. The utility of UPCR has not been formally evaluated. Our objective is to describe the diagnostic utility of UPCR by comparing Cx and UPCR results, and their impact on management.

**Figure 1:**
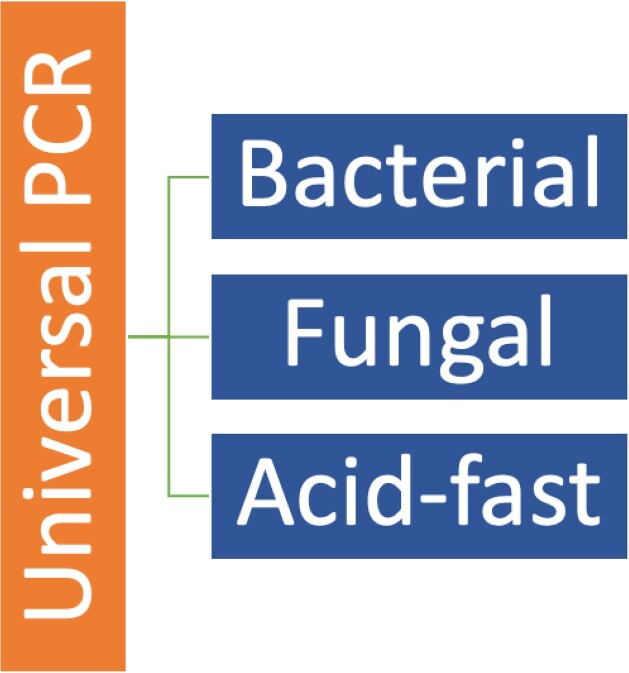
Introduction - Types of Universal PCR Testing

**Methods:**

We retrospectively collected data on UPCR and culture results and changes in antimicrobial therapy based on UPCR results for all patients with at least 1 UPCR test done during the 2-year study period.

**Results:**

367 UPCR tests were performed over 24 months on 155 patients. From 367 tests, 119 were FUPCR, 111 AFUPCR, and 137 BUPCR.

32/155 (20.6%) patients had positive UPCR. 25/32 were BUPCR and 7/32 were FUPCR. No AFUPCR was positive.

In 8/155 (5.2%) patients management was changed based on UPCR results:

- Positive UPCR results directed treatment in 5 patients: 4 patients had positive UPCR and negative culture, and 1 had both UPCR and Cx positive but for different organisms. In all 5 therapy was changed in favor of UPCR result. All 5 tests were BUPCR.
- Negative UPCR led to antimicrobial discontinuation in 3 patients.

11/155 (7.1%) patients had negative UPCR and positive Cx, 10 of which were BUPCR and 1 AFUPCR. These results did not change management.

**Figure 2:**
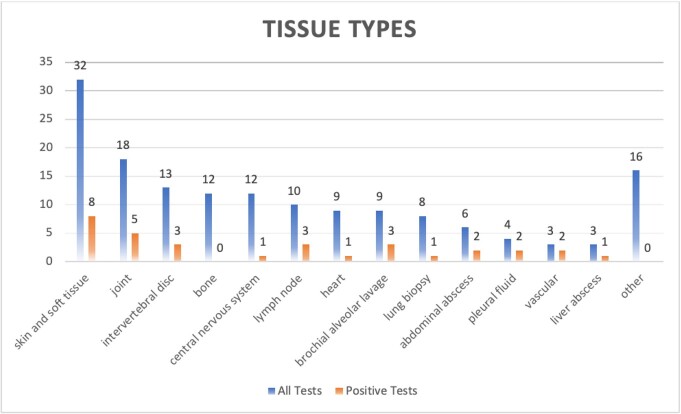
Results – Type of Tissue and Test Results

**Figure 3:**
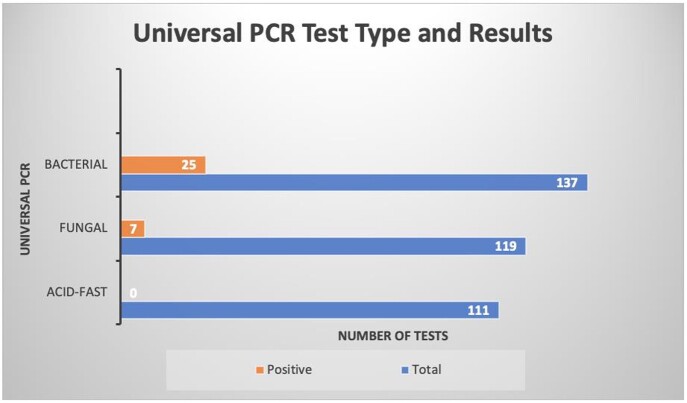
Results – Universal PCR Test Type and Results

**Figure 4:**
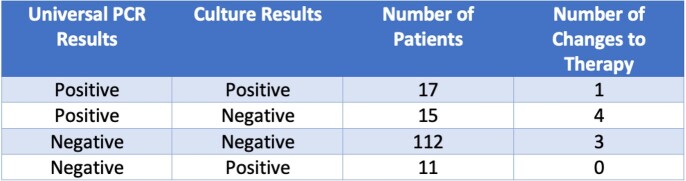
Results - Summary

**Conclusion:**

Based on the real-world experience, UPCR results have limited impact on antimicrobial management in our institution. Further studies may try to identify clinical scenarios where UPCR may be of better clinical utility.

**Disclosures:**

**All Authors**: No reported disclosures.

